# Single‐cell transcriptomics implicates the FEZ1–DKK1 axis in the regulation of corneal epithelial cell proliferation and senescence

**DOI:** 10.1111/cpr.13433

**Published:** 2023-02-27

**Authors:** Liqiong Zhu, Li Wang, Dongmei Liu, Chaoqun Chen, Kunlun Mo, Xihong Lan, Jiafeng Liu, Ying Huang, Dianlei Guo, Huaxing Huang, Mingsen Li, Huizhen Guo, Jieying Tan, Kang Zhang, Jianping Ji, Jin Yuan, Hong Ouyang

**Affiliations:** ^1^ State Key Laboratory of Ophthalmology, Zhongshan Ophthalmic Center Sun Yat‐sen University, Guangdong Provincial Key Laboratory of Ophthalmology and Visual Science| Guangzhou China; ^2^ Center for Biomedicine and Innovations, Faculty of Medicine Macau University of Science and Technology China

## Abstract

Limbal stem/progenitor cells (LSC) represent the source of corneal epithelium renewal. LSC proliferation and differentiation are essential for corneal homeostasis, however, the regulatory mechanism remains largely unexplored. Here, we performed single‐cell RNA sequencing and discovered proliferation heterogeneity as well as spontaneously differentiated and senescent cell subgroups in multiply passaged primary LSC. Fasciculation and elongation protein zeta 1 (FEZ1) and Dickkopf‐1 (DKK1) were identified as two significant regulators of LSC proliferation and senescence. These two factors were mainly expressed in undifferentiated corneal epithelial cells (CECs). Knocking down the expression of either FEZ1 or DKK1 reduced cell division and caused cell cycle arrest. We observed that DKK1 acted as a downstream target of FEZ1 in LSC and that exogenous DKK1 protein partially prevented growth arrest and senescence upon FEZ1 suppression in vitro. In a mouse model of corneal injury, DKK1 also rescued the corneal epithelium after recovery was inhibited by FEZ1 suppression. Hence, the FEZ1–DKK1 axis was required for CEC proliferation and the juvenile state and can potentially be targeted as a therapeutic strategy for promoting recovery after corneal injury.

## Graphical abstract

We isolated and cultured the limbal stem/progenitor cells (LSC) from donor human corneas. To comprehensively investigate LSC proliferation heterogeneity, we performed single‐cell RNA sequencing in multiply passaged LSC, and identified its diverse potential to proliferate, differentiate, or become senescent. The comparison analysis revealed FEZ1–DKK1 axis in the regulation of LSC proliferation and senescence. Upon in vitro LSC cultured system and in vivo mouse corneal wound healing model, we clarified that FEZ1 sustained LSC proliferation ability and suppressed senescence by targeting its downstream regulator DKK1.

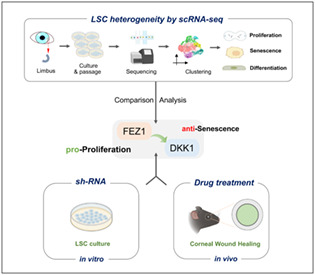

## 1 INTRODUCTION

The corneal epithelium is the outermost cell layer of the anterior cornea, and is essential for maintaining corneal homeostasis and transparency. The integrity and renewal of the corneal epithelium are sustained by limbal stem/progenitor cells (LSC) that reside in the peripheral limbus.^1^ During corneal epithelium turnover or corneal injury, LSC become activated, migrate centripetally, and undergo simultaneous proliferation, differentiation, and stratification processes to regenerate a functional corneal epithelium. Dysfunctional LSC may fail to help sustaining the epithelium regeneration to maintain or restore vision.^2^

Proliferation and differentiation are fundamental for adult stem cells to help maintaining tissue homeostasis and promote wound healing.^3^, ^4^ Upon differentiation, switches in the levels of specific keratins are observed in most epithelial tissues. For example, in the cornea, keratin 19 (KRT19)‐ and KRT15‐positive cells are mainly located in the limbus, whereas the central corneal epithelium is defined by markers of a mature corneal epithelium (KRT3 and KRT12).^1^, ^5^ As found in other stratified squamous epithelial cells, p63 (a well‐established transcription factor) is responsible for the self‐renewal of LSC. Shalom‐Feuerstein et al.^6^ reported that p63‐deficient mice exhibited disrupted corneal epithelial cell (CEC) differentiation and stratification. Recently, several factors have been implicated in LSC expansion in vitro, including basal cell adhesion molecule,^7^ tetraspanin 7,^8^ and SRY‐box transcription factor 2,^9^ however, the underlying regulatory mechanism of LSC proliferation remains largely unclear.

Various approaches have been used to explore the mechanism of epithelial cell proliferation, including transgenic mouse models,^6^ lineage‐tracking technology,^1^, ^10^ holoclone formation in cell culture,^11^ and three‐dimensional^5^ or air‐lifting^7^ corneal–epithelial differentiation systems. It is worth noting that epithelial stem/progenitor cells exhibit diverse proliferation capacities and an overall proliferation decline during cell division in vitro. Spontaneous differentiation and replicative senescence are also observed during this process.^12^, ^13^, ^14^ Thus, we decided to study LSC proliferation heterogeneity in a population of multiply passaged primary LSC by performing single‐cell RNA sequencing (scRNA‐seq), a useful method for uncovering cell subsets and cellular heterogeneity.^15^, ^16^

Here, we studied the cellular states of a population of multiply passaged LSC with diverse potentials to proliferate, differentiate, or become senescent. Fasciculation and elongation protein zeta 1 (FEZ1) and Dickkopf‐1 (DKK1) were identified as effective regulators of the maintenance of LSC proliferation and the suppression of LSC senescence. DKK1 functioned downstream of FEZ1 and rescued the growth arrest of cultured LSC and wound healing in a mouse model of corneal injury. Our work provides an in‐depth understanding of the dynamic changes in the cellular states of LSC and the regulatory pathway involved in sustaining their proliferation potential.

## 2 MATERIALS AND METHODS

### 2.1 Cell culture

Human LSC was obtained from limbal tissues, which were obtained from the eye bank of the Zhongshan Ophthalmology Center (2020KYPZ115) as described in the ethics statement. The cell suspension was prepared as follows: The limbal segments were cut into pieces and digested with 0.2% Collagenase IV solution (Gibco) for 2 h at 37°C. Next, the cell solution was further digested with 0.25% trypsin–EDTA (Gibco) for 15 min. LSC was cultured on 2% Matrigel‐coated polystyrene plates (Corning). The culture medium was prepared as previously described.^5^

To obtain CEC, LSC were seeded on 30 μg/mL Collagen I (Gibco)‐coated transwell plates (Corning) with the cell density of 2.5 × 10^5^ cells per insert. LSC medium was changed every other day until cells reached 100% confluence. For differentiation, the medium in the upper insert was removed. The volume of medium in the lower compartment was reduced to 200 μL per well and changed every day for another 5 days. All reagent details in this study are listed in Table S1.

### 2.2 Transfection

Short‐hairpin RNAs (shRNAs) specific targeting *FEZ1* and *DKK1* were inserted into PLKO.1 plasmid. A non‐target *scramble*‐shRNA (Addgene) was used as the negative control. Two individual shRNA were designed for each gene and used separately. LSC were infected with lentivirus in the presence of 8 μg/mL polybrene for 36 h, followed by selection with 2 μg/mL puromycin for 2 days. The shRNA sequences are shown in Table S2.

### 2.3 RNA extraction and quantitative polymerase chain reaction

Total RNA was obtained using the RNeasy kit (Tiangen) and then converted into cDNA with the PrimeScriptTM RT Master Mix Kit (Takara). Quantitative reverse transcription polymerase chain reaction (qRT‐PCR) was performed by mixing appropriate primers, cDNA, and iTaq Universal SYBR Green Supermix kit (Bio‐Rad). Data were analysed using a QuantStudio 7 Flex system (Life Technologies) and normalized by GAPDH. Experiments were carried out in triplicates. The primers are listed in Table S3.

### 2.4 scRNA‐seq sample preparation

Single cells were isolated from limbus tissue. Limbus sample was digested and then cultured in vitro for five passages (P5). The P5 LSC cells were isolated and 3′‐end sequencing was performed by CapitalBio using the 10× Genomics's protocol.

### 2.5 scRNA‐seq data processing

Sequencing reads alignment, barcode counting, UMI counting and genome assignment (hg19) were processed using Cell Ranger default settings pipeline (10× Genomics). Gene expression levels were normalized and scaled by factor of 10,000. The downstream clustering and differential expression analyses were performed using Seurat R package. Following parameters were applied to exclude low‐quality cells or genes: genes detected in <3 cells, cells with <200 or >10,000 expressed genes or >10% mitochondrial UMI counts. Nineteen thousand five hundred twenty genes across 4293 cells were eventually revealed.

FindClusters and FindNeighbors functions of Seurat were used to generate six clusters with top 15 principal components (PCs) and a resolution of 0.25. The unsupervised uniform manifold approximation and projection (UMAP) was applied for data display, FindAllMarkers function in Seurat was used to identify differentially expressed marker genes in each cluster. Nebulosa R package was used for gene density distribution display. Trajectory analysis was performed by two methods, Monocle2 pipeline with ordering gene set fit the following parameters: mean_expression ≥0.5 and dispersion_empirical ≥1, and PHATE pipeline with the following parameters: knn = 5, decay = 1, t = 10 for phate function and t = 4 for magic function. BEAM function in Monocle2 was applied to uncover the differential expression genes between Monocle trajectory branch.

Cell cycle scoring was estimated using Seurat's CellCycleScoring function with cell cycle phase marker genes, and cells were then assigned to G1, S, or G2/M phase. Seurat's AddModuleScore function and cell aging related gene cohort (GO:0007568) were used for aging score estimation. Cellchat R package was applied to infer cell–cell clusters communications between clusters.

### 2.6 RNA sequencing and data processing

After total RNA extraction, RNA sequencing (RNA‐seq) libraries were generated by TruSeq Stranded mRNA Library Prep kit (Illumina) and sequenced using paired‐end 150 reads setting on Novaseq 6000 S4 platform. STAR (version 2.6.1a) was used for mapping sequencing reads to human genome(hg19). Transcripts per million reads values were called using RSEM (version v1.3.0). DESeq2 (version 1.20.0) software was used to identify the differential expression genes, with *p* < 0.01 and log2 fold change >1. The differential expression genes were then uploaded to the online functional analysis tool The Database for Annotation, Visualization and Integrated Discovery (https://david.ncifcrf.gov/home.jsp) for performing Gene Ontology biological process (GO BP) enrichment and Kyoto Encyclopedia of Genes and Genomes (KEGG) analysis. Gene set enrichment analysis (GSEA) was performed with KEGG gene sets. The significance of the data was assessed by false discovery rate *q* ≤ 0.05. The results were plotted with ggplot2 package.

### 2.7 Haematoxylin and eosin and immunofluorescence staining

Deparaffinized slides were stained with haematoxylin and eosin (H&E) solution. Four percent paraformaldehyde (PFA)‐fixed cells or 10% neutral‐buffered formalin‐fixed tissue sections were permeabilized with 0.3% Triton X‐100 for 15 min, followed by 3% BSA blocking for 1 h. The samples were next incubated overnight at 4°C with specific primary antibodies. After washing with phosphate‐buffered saline (PBS), appropriate secondary antibodies were treated for 2 h at room temperature. Cell nuclei were then stained with Hoechst 33342 (Invitrogen). Samples were photographed with a ZEISS LSM 800 confocal microscope. The antibodies are listed in Table S4.

### 2.8 CFSE assay

5,6‐Carboxyfluorescein diacetate succinimidyl ester (CFSE) cell proliferation kit was used to determine the cell growth ability. Cells were labelled with 3 μM CFSE staining solution at 37°C for 15 min. After staining, cells were seeded into six‐well plates with the same amount, and cultured for another 8 days. Meanwhile, some labelled‐cells were immediately fixed to measure the original CFSE fluorescence intensity. Afterward, the cell proliferation ability was examined by flow cytometry (BD LSRFortessa Cell Analyser).

### 2.9 Senescence‐associated β‐galactosidase staining

Senescence‐associated β‐galactosidase (SA‐β‐gal) staining was performed using an SA‐β‐gal staining kit (Beyotime). All steps were according to the manufacturer's standard protocol. The percentage of positive staining cells was quantified over four random fields.

### 2.10 Cell cycle analysis

After transfection, cells were digested and centrifuged at 1000*g* for 3–5 min, followed by washing with cold PBS for one time. Cells were then fixed for 12–24 h with 70% ethanol at 4°C. Cell suspension was centrifuged at 1000*g* for 5 min and washed with cold PBS for one time. Cells were harvested, resuspended, and stained with 25 μg/mL propidium iodide and 100 μg/mL RNase A for 30 min at 37°C. Samples were analysed with flow cytometry (BD LSRFortessa Cell Analyser) within 24 h with 10,000 events in each condition.

### 2.11 MitoTracker staining

MitoTracker staining was performed using a MitoTracker Red CMXRos kit (Cell Signalling Technology). Samples were incubated in 50 nM MitoTracker staining solution for 30 min at 37°C. Cells were washed with PBS and then fixed with cold methyl alcohol for 15 min at room temperature. Cells were washed again with PBS, stained with Hoechst 33342, and then proceeded with the photograph procedure.

### 2.12 Crystal violet staining assay

5 × 10^4^ Cells/well were seeded into six‐well plates with Collagen I pretreated. LSC was divided into three groups: *scramble* (as control), *shFEZ1* (*FEZ1*‐depleted LSC group), *shFEZ1* + rhDKK1 (the rescue group with recombinant human DKK1 [rhDKK1]). Cells were cultured for 12–24 h to allow attachment, followed by exposure to different treatment media. Cells were transfected with lentivirus according to the steps described above. After selection with puromycin, cells were cultured under the treatment media for 5–8 days until the cell density of the control group reached 80%–90% confluence. After washing with PBS, the cells were fixed with 4% PFA at room temperature. Fifteen minutes later, the cells were washed with PBS and further stained with 0.5% crystal violet solution (Solarbio). After 20‐min incubation, all samples were washed with tap water, air‐dried overnight and photographed.

### 2.13 Mouse corneal epithelium defect model and treatments

C57BL/6 mice (6–8 weeks old) were used in this study. Mice were anaesthetised with sodium pentobarbital (50 mg/kg, intraperitoneal injection). The corneal epithelial wounding model was created as follows: The whole corneal epithelium was scraped off using the Algerbrush II corneal rust ring remover (Alger Co.), without encroaching on corneal stroma and limbus. Mice were divided into three groups: IgG (0.2 μg/μL, as control); FEZ1 antibody (0.2 μg/μL); FEZ1 antibody (0.2 μg/μL) + recombinant mouse DKK1 (rmDKK1) protein (10 ng/μL). After scraping off the corneal epithelium, differential treatments were performed by subconjunctival injection with a total injection volume of 4 μL. All animals were injected one time a day for 4 days. The epithelial defects were evaluated using sodium fluorescein staining, while the positive staining areas were calculated by ImageJ software (National Institution of Health).

### 2.14 Statistical analysis

Data are expressed as the mean ± SD. Student's *t*‐test was used to evaluate the difference between groups. *p* < 0.05 was considered as a significant difference.

## 3 RESULTS

### 3.1 scRNA‐seq revealed proliferation and differentiation heterogeneity in cultured LSC

**FIGURE 1:**
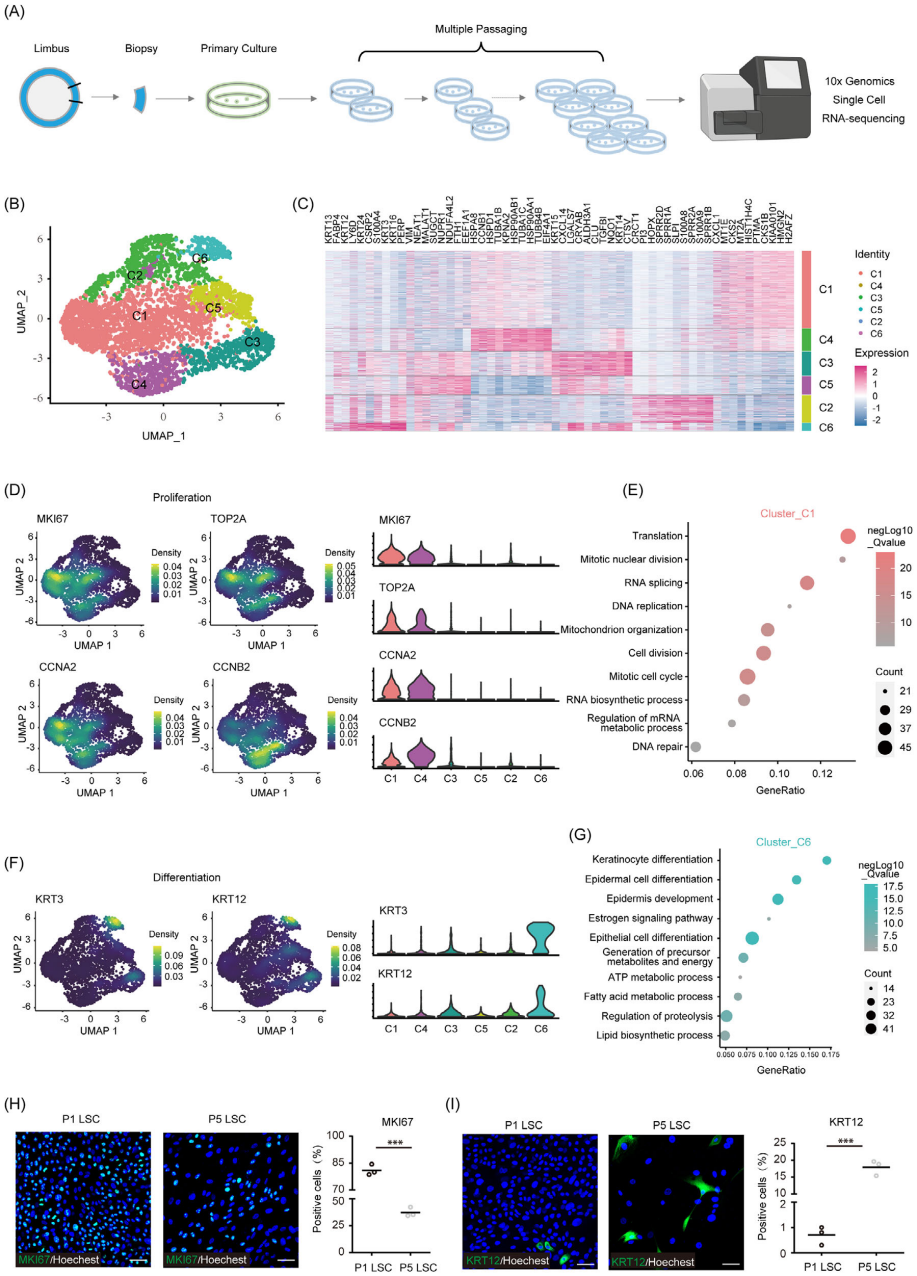
Single‐cell RNA sequencing identified the differentiation and proliferation subgroups in cultured limbal stem/progenitor cell (LSC). (A) Sample preparation pipeline for the single‐cell RNA sequencing of multiple‐passaged cultured LSC. (B) Uniform manifold approximation and projection plot presentation of cultured LSC. (C) Heatmap of top 10 marker genes for six clusters. (D, F) Density distribution and grouped expression plots of proliferative genes MKI67, TOP2A, CCNA2 and CCNB2, and epithelial cell differentiation genes KRT3 and KRT12. (E, G) Gene ontology analysis of marker genes of cluster C1 and C6. (H) Immunofluorescence staining and quantification of MKI67 in cultured passage 1 (P1) and P5 LSC (*n* = 3). (I) Immunofluorescence staining and quantification of KRT12 in cultured P1 and P5 LSC (*n* = 3). Scale bars = 50 μm. ***p < 0.001.

To explore spontaneous cellular heterogeneity and regulatory changes occurring in LSC at the transcriptome level, we analysed cultured LSC by scRNA‐seq. After dissecting and digesting healthy limbal tissues, primary LSC was cultured to confluence and then passaged five times before being subjected to 10× Genomics‐based scRNA‐seq (Figure 1A). We recovered 4293 single cells from mixed samples after filtering using the following parameters: minimum cells, minimum features, and mitochondrial gene percentile. The top 15 PCs were used to cluster cells with similar gene expression profiles. All cells were classified into six clusters (C1–C6) and visualized by UMAP (Figure 1B). Clusters C1 consisted of the majority of the cell composition while the C6 was the minority (Figure S1A). Differential gene expression analysis and whole‐transcriptome correlation analysis revealed three groups of clusters with similar gene expression profiles: C1/C4, C3/C5, and C2/C6 (Figure S1B).

To clarify the heterogeneity of the LSC clusters, marker genes in each cluster were obtained using the “FindAllMarkers” function of the Seurat package. The top 10‐ranking markers heatmap demonstrates distinct signatures of each cluster (Figure 1C). Cells in clusters C1 and C4 showed high expression of genes involved in cell cycle progression including H2AFZ, CKS1B, and CCNB1. In clusters C2 and C6, the cells showed high expression of genes involved in epithelial cell differentiation, including those encoding keratin and members of the SPRR gene family. Notably, the CEC markers KRT3 and KRT12 were highly coexpressed in cluster C6, suggesting that the cells were terminally differentiated. The UMAP density distribution and grouped expression of known proliferative genes (MKI67, TOP2A, CCNA2, and CCNB2) further confirmed that the cells in clusters C1 and C4 were actively proliferating (Figure 1D). In addition, KRT3 and KRT12, which are established corneal epithelial markers, were highly expressed exclusively in cluster C6, which confirmed the differentiated state (Figure 1F). GO analysis of marker genes revealed the proliferating and differentiated states of cells in clusters C1 and C6, respectively. The marker genes of cluster C1 were mainly enriched for proliferation‐associated GO terms including ‘Translation’, ‘mitotic nuclear division’, and ‘DNA replication’ (Figure 1E), whereas the marker genes of cluster C6 were mainly enriched for GO terms associated with epithelial cell differentiation including ‘keratinocyte differentiation’ ‘epidermal cell differentiation’, and ‘epithelial cell differentiation’ (Figure 1G). The marker genes of rest clusters C3 and C5 were mainly enriched for metabolic processes but not relevant to proliferation or differentiation (Figure S1C).

To further dissect the LSC differentiation and development progress of the clusters, trajectory analysis was performed using the Monocle2 and PHATE packages. Both the Monocle and PHATE pseudo‐temporal trajectories originated from the proliferating subclusters (C1 and C4) and terminated with the differentiated subcluster (C6) (Figure S1D,G). The cell portion distribution along the monocle pseudotime was shown in Figure S1F. The cells that expressed high levels of proliferation markers (MKI67, TOP2A, CCNA2, and CCNB2) mapped to the initiating region, whereas cells that expressed high levels of differentiation markers (KRT3 and KRT12) mapped to the end of the pseudotime trajectories (Figure S1E,H). To determine the cellular states along the Monocle trajectory, k‐means clustering was performed on the ordering genes, and four gene clusters were clustered according to the expression trends. GO analysis of the gene clusters revealed that cell proliferation‐related genes were enriched in the pseudotemporal early stage (Cluster 2), and epithelial differentiation‐related genes were enriched in the late stage (Cluster 1) (Figure S2A).

The Monocle trajectory bifurcated into two branches. Cluster C3 cells mainly comprised the minor branch (cell fate 1), whereas the proliferating and differentiated LSC were mainly located before the branch or in the major branch (cell fate 2) (Figure S2B). The marker genes of Cluster C3 were highly consistent with branch‐related genes, as revealed by the Monocle Beam analysis (Figure S2C). The overlapping genes were mainly enriched in metabolic processes, including aldehyde, pyruvate, vitamin, and monocarboxylic acid metabolism (Figure S2D), suggesting that Cluster C3 or the minor branch was isolated, mainly because of cell‐metabolism differences.

Immunofluorescence staining of MKI67 and KRT12 was performed to validate the proliferation and differentiation changes after LSC were passaged multiple times. With P5 LSC, the fraction of MKI67‐positive cells portion was significantly lower and the fraction of KRT12‐positive cells was significantly higher, when compared to those among P1 LSC (Figure 1H,I). The LSC‐specific markers PAX6 and p63 showed no significant alterations along with cell division (Figure S3A). These results demonstrate that the cultured LSC were highly proliferative but showed a steady decline in their proliferation abilities and spontaneous differentiation after multiple passages.

To quantitatively reveal the cell–cell communication networks within cell clusters, ‘CellChat’ package was adopted in LSC scRNA‐seq data. We found that the interactions number and strength were both much higher in the proliferative Cluster C1 and C4, but obviously lower in the differentiated Cluster C2 and C6 (Figure S4A,B), indicating the active transduction in proliferating cells. The dominant communication patterns were mainly enriched in genes related to WNT and FGF signalling pathway (Figure S4C,D). For WNT signalling communication, the interactions existed in all clusters but the differentiated Cluster C6 (Figure S4E,F,G), and WNT7B (FZD3/LRP5) ligand‐receptor (L‐R) pair contributed the major interaction power (Figure S4H). For FGF signalling communication, Clusters C1 and C4 were as the sender and differentiated C6 as the receiver (Figure S4E,I,J) while FGF5/FGF3R L‐R pair contributed the most interaction power (Figure S4K). The cell–cell communication analysis suggested that there may exist active WNT and FGF signalling interaction between proliferative and differentiated clusters.

### 3.2 scRNA‐seq identified the aging subgroup in cultured LSC

**FIGURE 2:**
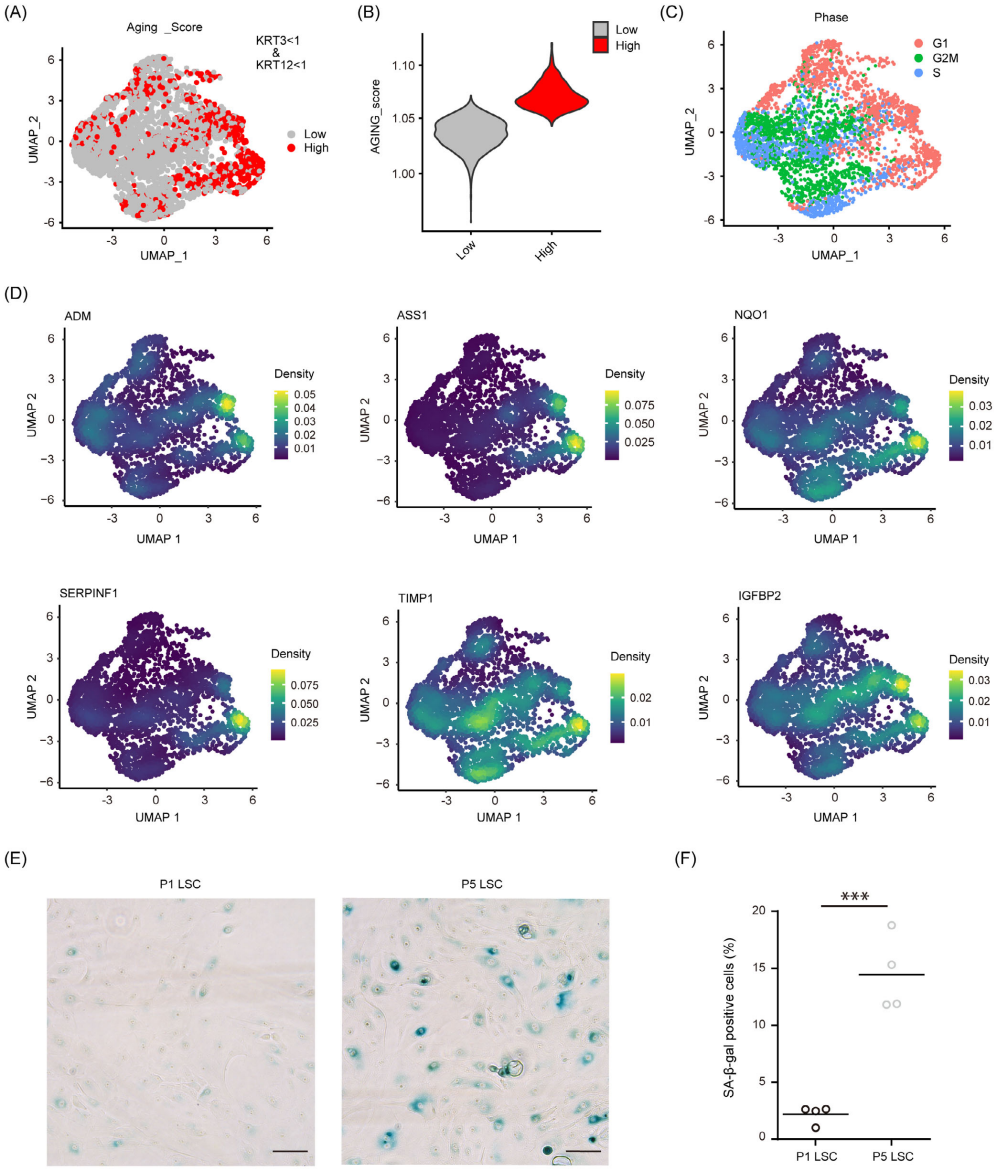
Single‐cell RNA sequencing identified the aging subgroup in cultured limbal stem/progenitor cell (LSC). (A, B) Aging scoring distribution and grouped expression plots. (C) Cell cycle stage plot based on cell cycle scoring. (D) Density distribution plot of aging‐associated genes including ADM, ASS1, NQO1, SERPINF1, TIMP1, and IGFBP2. (E, F) senescence‐associated β‐galactosidase staining and quantification in cultured passage 1 (P1) and P5 LSC (*n* = 4). Scale bars = 100 μm. ***p < 0.001.

In addition to undergoing spontaneous differentiation, in vitro‐cultured stem cells also tend to gradually become senescent or age after several passages. The core functions of stem cells decline with age. Therefore, we performed aging scoring to estimate the aging status based on the scRNA‐seq data. The differentiated subgroup (KRT3 > 1 and KRT12 > 1) was ignored before the analysis for a clearer distinction. The aging‐related gene cohort (GO:0007568) and ‘AddModuleScore’ function in the Seurat R package were used for aging scoring, cells with the top 25% aging scores were defined as ‘high cells’ and the rest were defined as ‘low cells’. A subgroup of cells had relatively high expression levels of aging‐related genes and, therefore, possessed a higher aging score (Figure 2A,B). Aged cells generally exited the cell cycle and were arrested in G1 phase. The cell cycle UMAP distribution (estimated using the ‘CellCycleScoring’ function) showed that cells in the subgroup with the higher aging score were consistently in G1 phase (Figure 2C). Some aging‐related genes were also distributed identically to the subgroup with the higher aging score, including ADM, ASS1, NQO1, SERPINF1, TIMP1, and IGFBP2 (Figure 2D). Staining for SA‐β‐gal showed significantly more SA‐β‐gal‐positive cells among P5 LSC than among P1 LSC (*p* < 0.001) (Figure 2E,F). These results suggest that multiple passages increased the senescent cell population in cultured LSC.

### 3.3 DKK1 and FEZ1 may participate in maintaining the proliferation of cultured LSC

**FIGURE 3:**
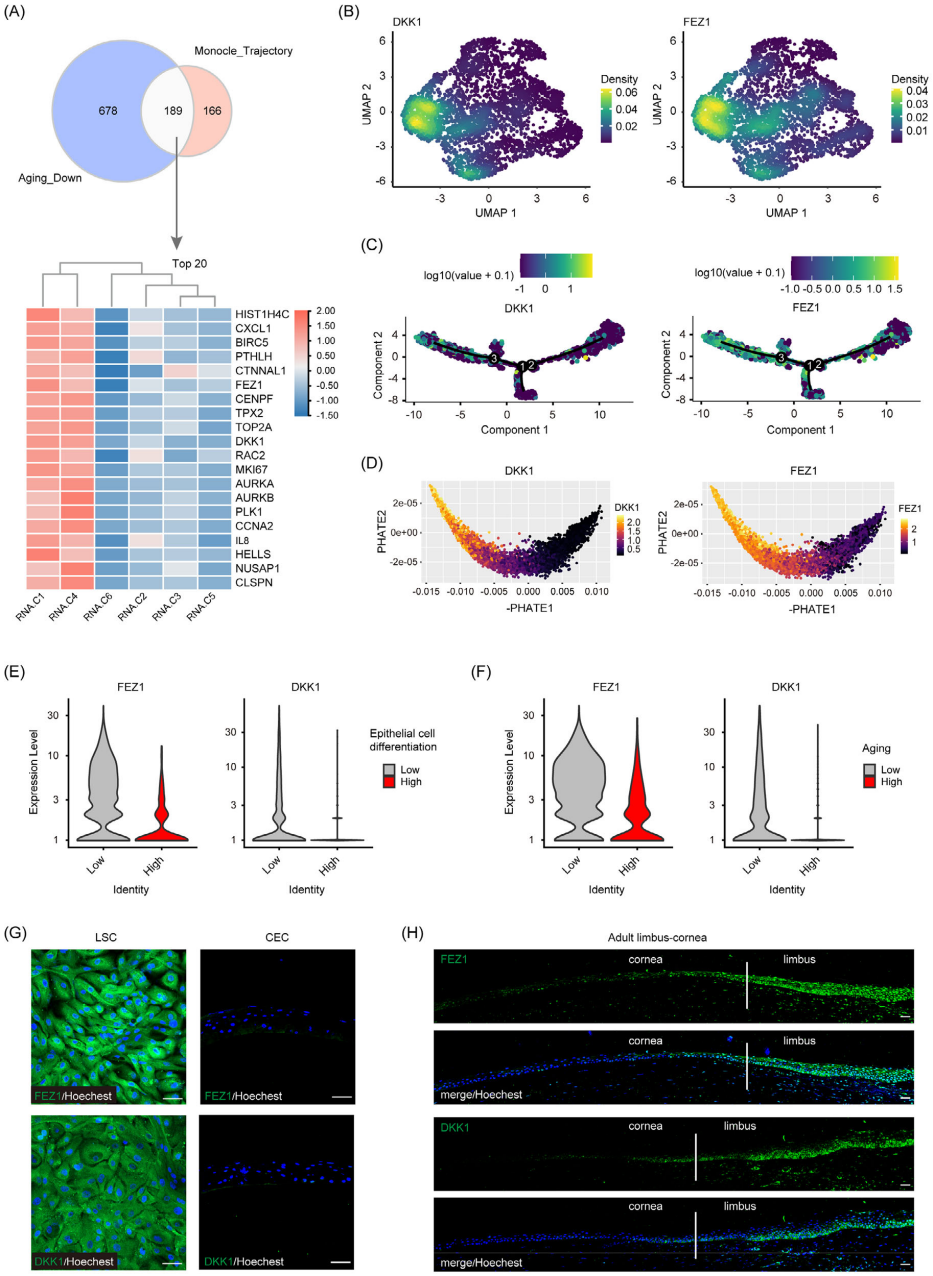
Dickkopf‐1 (DKK1) and fasciculation and elongation protein zeta 1 (FEZ1) may participate proliferative ability maintenance in cultured limbal stem/progenitor cell (LSC). (A) Overlapping of genes declined along trajectory and marker genes of the low‐aging score cells shown by Venn diagram and top 20 overlapping genes grouped expression by heatmap. (B) Density distribution plots of DKK1 and FEZ1. (C) DKK1 and FEZ1 mapping on Monocle trajectory. (D) DKK1 and FEZ1 mapping on PHATE trajectory. (E) FEZ1 and DKK1 grouped expression in high‐ or low‐differentiated cell groups. (F) FEZ1 and DKK1 grouped expression in high‐ or low‐aging score cell groups. (G) Immunofluorescence staining of FEZ1 and DKK1 in LSC and differentiated corneal epithelial cells. (H) Immunofluorescence staining of FEZ1 and DKK1 in adult human limbus‐cornea tissue. Scale bars = 50 μm.

We aimed to identify key regulatory genes responsible for the decreased proliferative ability and increased aging when the LSC was passaged multiple times. Therefore, the genes that showed decreased expression along the Monocle trajectory were selected and intersected with marker genes of the subgroup with the low‐aging score. The genes with the top 20 *q* values among the intersected 189 genes are presented for each single‐cell cluster in a heatmap (Figure 3A). After excluding genes that either had low expression or had been validated for proliferation or aging, DKK1 and FEZ1 were chosen for subsequent studies.

The cell populations that showed high DKK1 or FEZ1 expression only colocalized with the actively proliferative cluster (C1), as determined by performing UMAP density distributions (Figure 3B). Cells that expressed high levels of DKK1 and FEZ1 also explicitly mapped to the initial regions of both Monocle and PHATE trajectories, and DKK1 and FEZ1 expression gradually decreased along the pseudotime (Figure 3C,D). Combined with our findings that C1 cells were actively proliferating and that the proliferative ability decreased along the pseudotemporal trajectories, our results collectively suggest that DKK1 and FEZ1 may be involved in decreased proliferative capacity that occurred with LSC multiple‐passaging. DKK1 and FEZ1 were also highly expressed in the undifferentiated cell population and the cell population with a low‐aging score (Figure 3E,F), suggesting that both genes may regulate stemness or maintenance of the proliferation ability.

Next, we experimentally validated the corneal expression of DKK1 and FEZ1 in vitro and in vivo. CECs differentiated from LSC barely expressed DKK1 and FEZ1, whereas both two genes were highly expressed in the ancestor LSC (Figure 3G). In adult human limbal and corneal tissues, DKK1 and FEZ1 were mainly expressed in LSC‐located limbal region, but not in the central corneal epithelium (Figure 3H). These results demonstrated that DKK1 and FEZ1 may act as key regulators of proliferation, differentiation, and senescence in LSC.

### 3.4 LSC proliferation was altered after FEZ1 and DKK1 depletion

**FIGURE 4:**
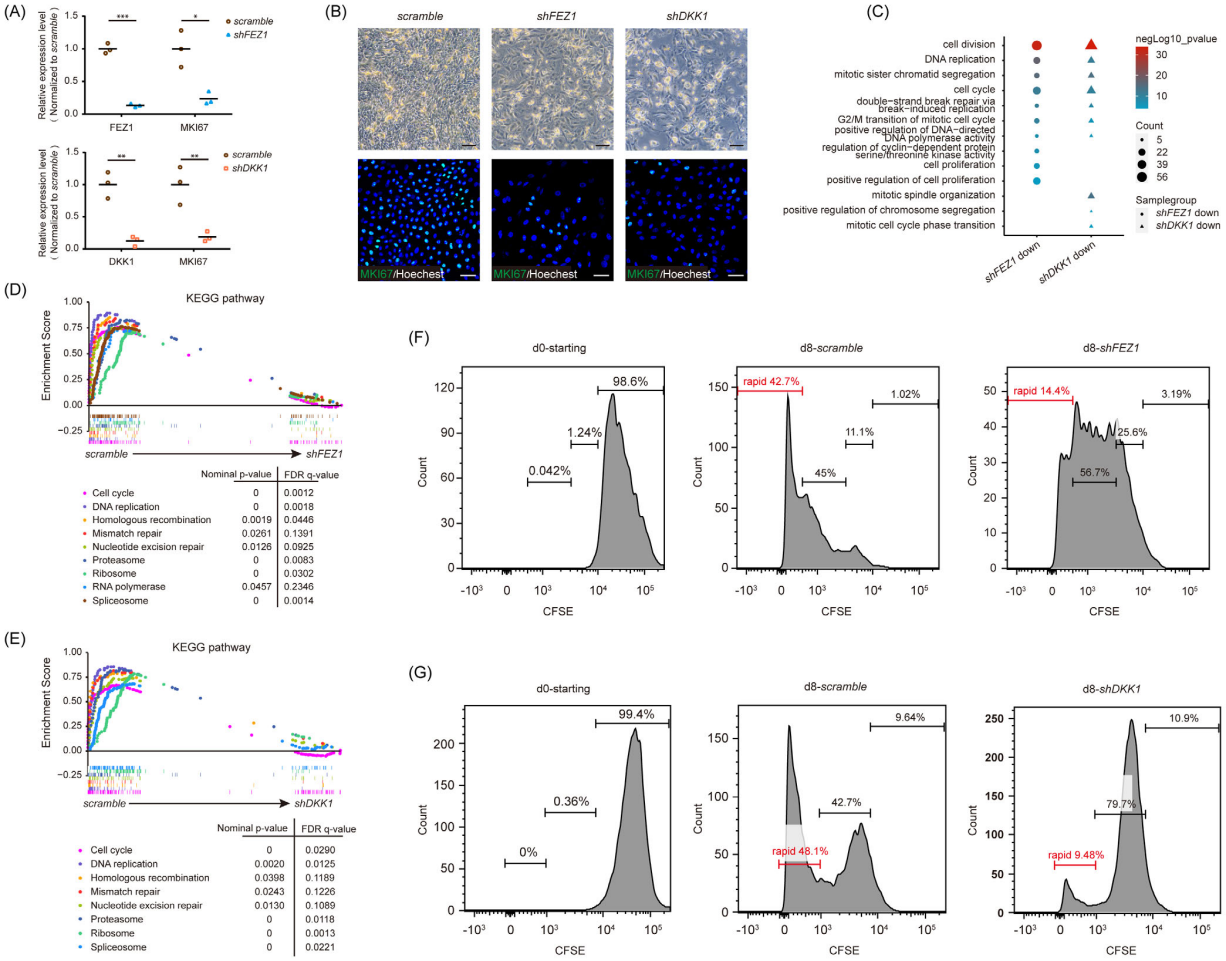
Loss of fasciculation and elongation protein zeta 1 (FEZ1) or Dickkopf‐1 (DKK1) results in growth arrest in limbal stem/progenitor cell (LSC). (A) Quantification of *FEZ1*, *DKK1*, and *MKI67* expression level in sh*FEZ1*‐ and sh*DKK1*‐LSC (*n* = 3). (B) Phase‐contrast images (upper panel) and immunostaining images (lower panel) of the LSC treated with *scramble*, *FEZ1*, and *DKK1* short‐hairpin RNA (shRNA). Scale bars = 200 μm (phase‐contrast images); 50 μm (immunostaining images). (C) Enriched Gene Ontology terms of the downregulated genes in *shFEZ1*‐ and *shDKK1*‐LSC. (D, E) Gene set enrichment analysis of proliferation‐related pathways in *shFEZ1*‐ (D) and *shDKK1*‐ (E) LSC. (F) The cell division frequency of 5,6‐carboxyfluorescein diacetate succinimidyl ester (CFSE)‐labelled LSC treated with *scramble* and *FEZ1* shRNA on Day 0 and Day 8. (G) The cell division frequency of CFSE‐labelled LSC treated with *scramble* and *DKK1* shRNA on Day 0 and Day 8 after staining. *p < 0.05; **p < 0.01; ***p < 0.001.

To further validate the function of FEZ1 and DKK1 in LSC, we specifically knocked down the expression of *FEZ1* or *DKK1* using shRNAs (*shFEZ1* or *shDKK1*, respectively). As expected from our scRNA‐seq analysis, knocking down *FEZ1* or *DKK1* in LSC resulted in lower cell densities and substantial MKI67 suppression, both at the mRNA and protein levels (Figure 4A,B). Transcriptome sequencing (RNA‐seq) was performed to identify *FEZ1*‐ and *DKK1*‐regulated gene signatures. GO analysis revealed that the genes that were downregulated by *FEZ1* or *DKK1* depletion were mainly enriched for terms associated with cell division, cell cycle, chromosome segregation, and cell proliferation. In terms of BPs, the GO terms were mainly enriched for those related to replication and cell division (Figure 4C). GSEA also revealed positive correlations between the target genes (*FEZ1* or *DKK1*) and several cell proliferation‐related pathways, such as cell cycle, DNA replication, homologous recombination, and RNA polymerase (Figure 4D,E). These results suggest that FEZ1 and DKK1 functioned similarly in supporting LSC proliferation.

CFSE staining was performed to compare the number of cell divisions occurring with *shFEZ1*‐, *shDKK1*‐, and *scramble*‐shRNA‐transfected LSC on Days 0 and 8. Using a FACS‐based CFSE assay, we observed that 14.4% of the cells in a rapid growth cell subset (left panel) of the *shFEZ1*‐LSC group divided on Day 8, which was significantly less than that of the control group (42.7%) (Figure 4F). Similarly, 9.48% of a rapidly growing subset of *shDKK1*‐transfected LSC divided on Day 8, which was also significantly smaller than that of the *scramble* group (48.1%) (Figure 4G). Taken together, the data indicate that FEZ1 and DKK1 participated in maintaining the proliferative ability in cultured LSC.

### 3.5 *FEZ1* and *DKK1* depletion partially induced a senescence phenotype

**FIGURE 5:**
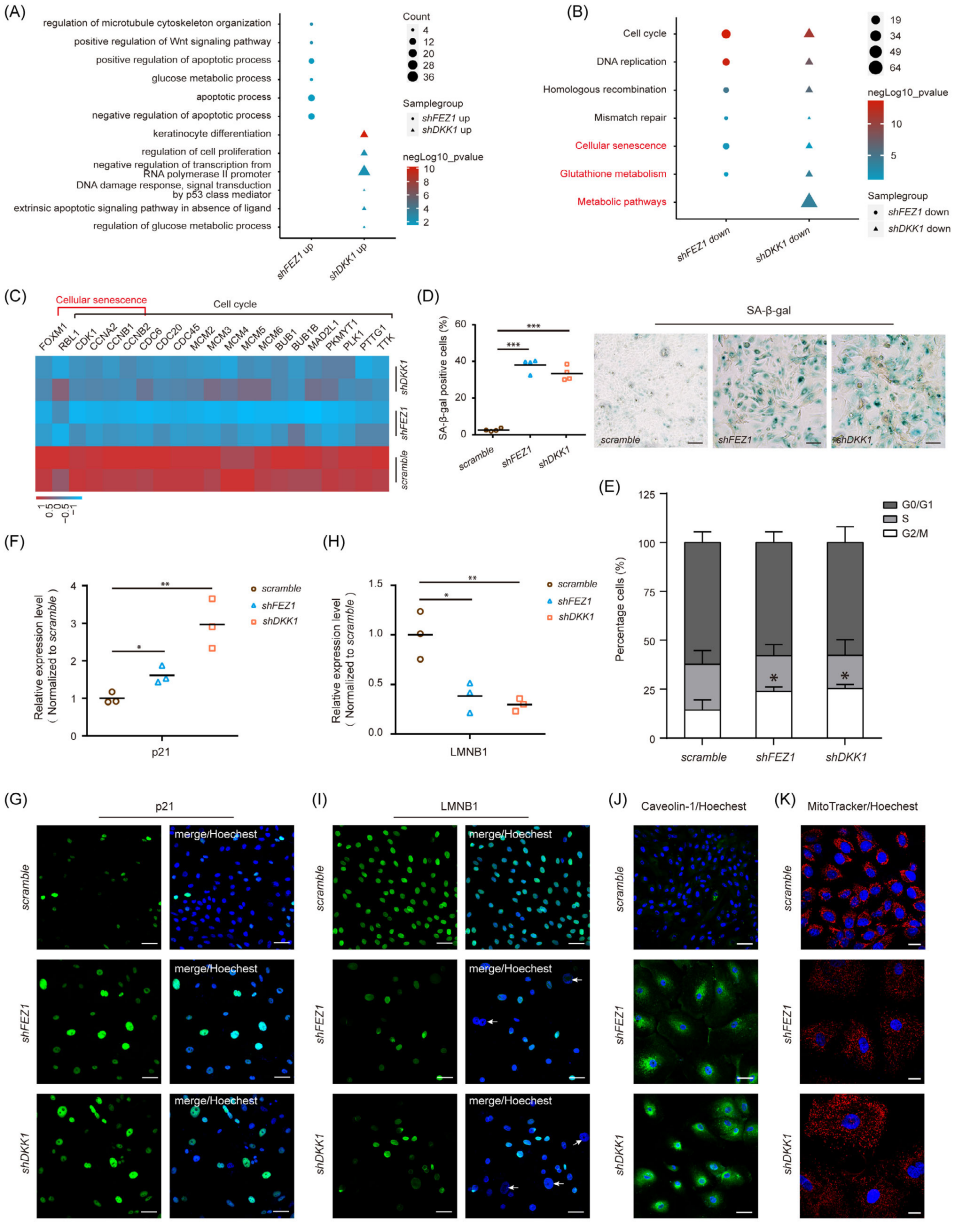
Fasciculation and elongation protein zeta 1 (FEZ1) and Dickkopf‐1 (DKK1) depletion partially induced senescence‐like features. (A) Enriched Gene Ontology (GO) terms of the upregulated genes in *shFEZ1*‐ and *shDKK1*‐ limbal stem/progenitor cell (LSC). (B) Enriched pathways of the downregulated genes in *shFEZ1*‐ and *shDKK1*‐LSC based on GO analysis. (C) Heatmap of the cellular senescence and cell cycle‐related genes in *scramble*, *sh‐FEZ1*‐ and *sh‐DKK1*‐LSC. (D) Quantification and representative images of senescence‐associated β‐galactosidase staining of *scramble*, *sh‐FEZ1*‐, and *sh‐DKK1*‐LSC (*n* = 4). Scale bars = 100 μm. (E) Bar plot showing the proportions of LSC in the difference cell cycle phases after *scramble*, *sh‐FEZ1*‐, and *sh‐DKK1*‐short‐hairpin RNA transfection (*n* = 3). Data are presented as mean ± SD. (F, H) Quantification of *p21* (F) and *LMNB1* (H) expression level in *scramble*, *sh‐FEZ1*‐, and *sh‐DKK1*‐LSC (*n* = 3). (G, I, J) Immunostaining images showing the changes of p21 (G), LMNB1 (I), and caveolin‐1 (J) expression level in *scramble*, *sh‐FEZ1*‐, and *sh‐DKK1*‐LSC. Scale bars = 50 μm. (K) MitoTracker pictures showing the dispersed distribution of mitochondria in *shFEZ1*‐ and *shDKK1*‐LSC. Scale bars = 20 μm. *p < 0.05; **p < 0.01; ***p < 0.001.

According to our scRNA‐seq analysis, FEZ1 and DKK1 might help maintain a balance between LSC proliferation and senescence. Therefore, we explored the participation of FEZ1 and DKK1 in LSC senescence. GO BP analysis of the upregulated genes in *shFEZ1*‐ and *shDKK1*‐transfected LSC showed main associations with apoptosis and glucose metabolism (Figure 5A). KEGG analysis showed that the downregulated genes in *shFEZ1*‐ and *shDKK1*‐transfected LSC were enriched for pathways associated with cell cycle, cellular senescence, and metabolic (Figure 5B). All of these BPs and pathways have been implicated in cellular senescence.^17^ Moreover, genes related to cellular senescence and cell cycle progression showed similar expression patterns in the *shFEZ1*‐ and *shDKK1*‐transfected LSC. Depletion of *FEZ1* and *DKK1* resulted in reduced expression of cellular senescence‐related genes, which regulate genes related to the ‘transcription of the cell cycle’ (FOXM1)^18^ or ‘transition of the cell cycle phase’ (RBL1).^19^ FEZ1 and DKK1 depletion also led to significantly decreased levels of genes related to cell cycle progression, including cyclin‐dependent kinases, cyclins,^20^ the pre‐replication complex (CDCs and MCMs),^21^ and spindle‐checkpoint components (BUBs and MAD2L1)^22^ (Figure 5C). These results suggest that *FEZ1* and *DKK1* depletion may suppress cell cycle progression and cell proliferation, which are characteristic features of cellular senescence.

The percentage of SA‐β‐gal‐positive cells increased by 14.7‐ to 12.9‐fold in *shFEZ1*‐ and *shDKK1*‐transfected LSC, respectively (Figure 5D). Because cell cycle arrest is recognized as an important feature of cellular senescence, cell cycle progression was assessed by flow cytometry. The control group was consisted of 62.2% G0/G1 phase, 23.5% S phase, and 14.3% G2/M phase cells. *FEZ1* and *DKK1* depletion did not affect the G0/G1 phase ratio (57.8 and 57.6%, respectively) or the S phase proportion (18.4 and 17%, respectively), but caused a notable cell cycle arrest in G2/M phase (23.8 and 25.4%, respectively) (Figure 5E). Expression of p21 (a core cell cycle inhibitory protein and senescence marker)^17^, ^23^ increased upon *FEZ1* and *DKK1* depletion at both mRNA and protein levels. Transcriptionally, *p21* mRNA increased 1.61‐ and 2.97‐fold in *shFEZ1*‐ or *shDKK1*‐transfected LSC, respectively (Figure 5F). Translationally, a substantially larger portion of p21‐positive cells was found among *shFEZ1*‐ and *shDKK1*‐transfected LSC than in *scramble*‐shRNA‐transfected LSC, and this increase was accompanied by irregular cell morphologies (Figure 5G).

Cellular senescence is a complex process involving disruption of the nuclear integrity, changes in the plasma membrane (PM) composition, and alterations in the mitochondrial membrane potential.^17^ *shFEZ1*‐ and *shDKK1*‐transfected LSC showed 2.6‐ and 3.4‐fold lower mRNA expression of *LMNB1*, which is an indicator of nuclear integrity^24^ (Figure 5H). The protein level of LMNB1 was also significantly lower in *shFEZ1*‐ and *shDKK1*‐transfected LSC than in *scramble*‐shRNA‐transfected LSC (Figure 5I). Intriguingly, cells with lower LMNB1 expression exhibited aberrantly enlarged nuclei (white arrows), which is a typical feature of senescent cells (Figure 5I). Moreover, *FEZ1* and *DKK1* depletion in LSC significantly increased the expression of caveolin‐1, an indicator of PM alterations induced by cellular senescence^25^ (Figure 5J). An abnormal distribution is an indication of unhealthy mitochondria and is an important feature of cellular senescence.^26^ By performing MitoTracker staining, we found that the mitochondria of control (*scramble*‐shRNA‐transfected) LSC were dense, fused, and had a perinuclear distribution. However, the mitochondria in *shFEZ1*‐ and *shDKK1*‐transfected LSC showed dispersed distributions and were in the fission state (Figure 5K), similar to senescent cells.^27^ Taken together, these data indicate that *FEZ1* and *DKK1* depletion induced multiple senescence phenotypes, demonstrating the participation of both genes in regulating LSC senescence.

### 3.6 FEZ1‐regulated LSC proliferation and senescence via FEZ1–DKK1 axis

**FIGURE 6:**
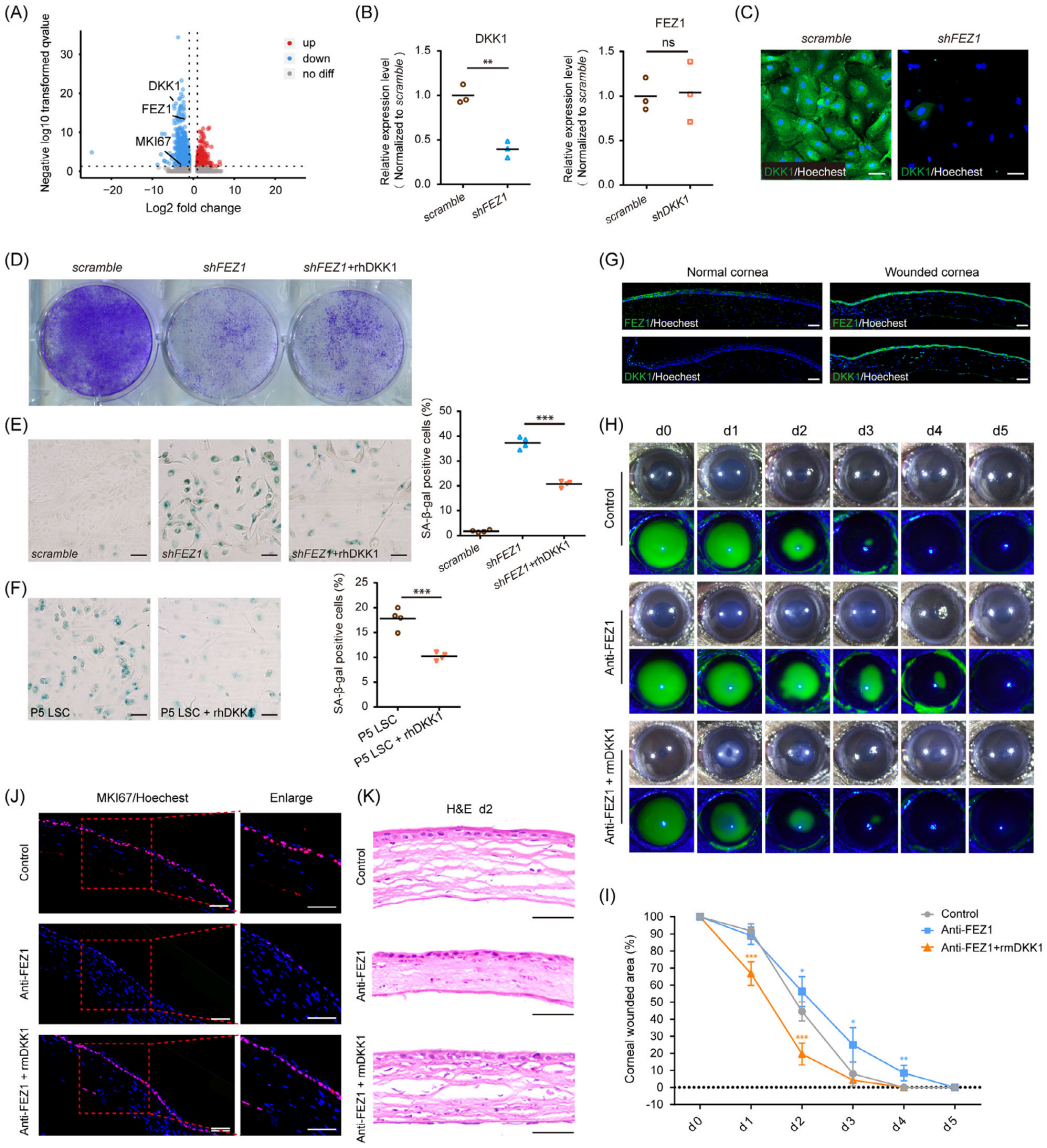
Exogenous Dickkopf‐1 (DKK1) partially rescues fasciculation and elongation protein zeta 1 (FEZ1) knockdown‐impaired cell growth and protects limbal stem/progenitor cell (LSC) from senescence. (A) Volcano plot showing the differential expressed genes in *shFEZ1*‐LSC versus the control. (B) Quantification of *DKK1* and *FEZ1* expression level in *shFEZ1*‐LSC versus the control or *shDKK1*‐LSC versus the control. (C) Immunostaining images showing the expression level of DKK1 in control or *shFEZ1‐*LSC. (D) Crystal violet images showing the *shFEZ1*‐LSC with or without recombinant human DKK1 (rhDKK1) treatment. (E) Senescence‐associated β‐galactosidase (SA‐β‐gal) staining and quantification of the *shFEZ1*‐LSC with or without rhDKK1 treatment (*n* = 4). (F) SA‐β‐gal staining and quantification of passage 5 LSC with or without rhDKK1 treatment (*n* = 4). (G) Immunostaining images showing the expression of FEZ1 and DKK1 in normal corneas and wounded corneas. (H) Representative images showing the corneal epithelial defects in mice cornea wound model treated with IgG (as control), FEZ1 antibody and FEZ1 antibody combined with recombinant mouse DKK1 (rmDKK1) (for each group: white light micrograph [upper panel], fluorescein staining images [lower panel]). (I) Quantification of epithelium regeneration in mice cornea with different treatments. The residual epithelial defect area was shown as the percentage of the current defect area versus the original defect area (*n* = 5). Data are shown as mean ± SD. (J) Immunostaining images showing the expression of MKI67 in the newly regenerated corneal epithelium in the peripheral corneal region with a different treatment on Day 2 after injury. (K) Haematoxylin and eosin (H&E) staining images of the newly regenerated corneal epithelium in the peripheral corneal region with a different treatment at 2 days after injury. Scale bars = 50 μm (immunostaining and H&E staining images); 100 μm (SA‐β‐gal staining images). *p < 0.05; **p < 0.01; ***p < 0.001.

Based on the similar effects of FEZ1 and DKK1 on LSC proliferation and senescence, we further investigated the relationship between FEZ1 and DKK1. RNA‐seq results showed that *FEZ1* depletion decreased the expression of both *MKI67* and *DKK1* (Figure 6A). Consistent with the RNA‐seq results, DKK1 expression diminished after *FEZ1* knockdown at both the mRNA and protein levels (Figure 6B,C); however, *DKK1* depletion did not alter *FEZ1* mRNA levels (Figure 6B), indicating that FEZ1 may act upstream of DKK1 in LSC.

Rescue experiments validated the upstream role of FEZ1, both in vitro and in vivo. rhDKK1 (100 ng/mL) protein was added to cultured LSC transfected with *shFEZ1* shRNA or control shRNA. Crystal violet staining and SA‐β‐gal staining were performed to assess the growth arrest and senescence phenotype, respectively. Crystal violet staining showed that growth arrest induced by *FEZ1* depletion was partially reversed by rhDKK1 treatment (Figure 6D). Moreover, rhDKK1 treatment significantly attenuated the *FEZ1*‐depletion induced SA‐β‐gal activity (Figure 6E) and replicative senescence in P5 LSC (Figure 6F). These results suggest that DKK1 functioned downstream of FEZ1 to regulate LSC proliferation and senescence.

To explore the downstream factors of FEZ1–DKK1 axis, GO BP analysis was applied to the 230 genes that were downregulated in both *shFEZ1*‐ and *shDKK1*‐transfected LSC cells. The co‐descending genes were mainly enriched in terms related to cell division, cell cycle progression, replication, and cell proliferation (Figure S5A). The expression of co‐descending genes, which were related to cell proliferation (FGFBP1, FOSL1, and MELK)^28^, ^29^, ^30^ or cell growth and cellular senescence (AURKB, FOXM1, HMGA2, and HMGB2),^31^, ^32^, ^33^, ^34^, ^35^ were further verified by qRT‐PCR. Results showed that these genes were assuredly decreased in *shFEZ1*‐ and *shDKK1*‐ transfected LSC (Figure S5B), suggesting the possible downstream regulators and mechanism of FEZ1–DKK1 axis in LSC proliferation.

To validate the functions of FEZ1 and DKK1 in vivo, corneal wound healing experiments were performed using a mouse model of corneal injury. Compared to the expression levels of FEZ1 and DKK1 observed in normal mouse corneas, those in regenerated epithelium tissues after wounding were both significantly higher (Figure 6G), implying that FEZ1 and DKK1 participated in corneal wound healing in vivo. To validate the associations between FEZ1 and DKK1 expression during corneal wound closure *in vivo*, we tested subconjunctival injection of rmDKK1 (10 ng/μL) and a FEZ1 antibody using a mouse model of corneal injury. The results showed that blocking FEZ1 caused a slight delay in corneal wound closure on Day 2, followed by a notable delay on Day 3, when compared to wound closure rates of the IgG‐injected (antibody control) group. Fluorescein sodium staining showed that blocking FEZ1 delayed complete wound healing by 1 day (8.46%). However, in the group cotreated with rmDKK1 and the FEZ1 antibody, remarkably smaller wound areas were observed (compared to those in the other two groups) on Day 1, followed by a similar healing pattern versus that in the control group (Figure 6H,I). Unwounded corneas showed no detectable alterations after FEZ1 antibody injection, as expected (Figure S6A).

Consistent with the fluorescein sodium staining results, the marked decrease in MKI67‐positive cells observed with FEZ1 did not occur after cotreatment with rmDKK1 and FEZ1 antibody (Figure 6J). In addition, H&E staining showed that the thinner‐epithelium phenotype observed after FEZ1 blocking did not occur in the cotreatment group on Day 2 (Figure 6K). The corneal epithelium showed no significant differences among the three groups after complete regeneration on Day 7 (Figure S6B,C). These results indicated that blocking endogenous FEZ1 delayed epithelial healing, which could be prevented by treatment with exogenous rmDKK1. Collectively, our findings indicate that FEZ1 participated in corneal wound healing through its downstream regulator, DKK1, in vivo.

## 4 DISCUSSION

The maintenance of the proliferation and differentiation capacities of adult stem cells is critical for tissue repair and homeostasis. Here, we discovered the proliferation, differentiation, and senescence heterogeneity in multiply passaged primary LSC and clarified the dynamic transcriptomic changes that occurred upon cell‐state switching. FEZ1 and its downstream target DKK1 were further identified as master regulators of proliferation and senescence, highlighting potential therapeutic applications in corneal wound healing.

scRNA‐seq is widely used to define distinct cell clusters within tissues. In a recent scRNA‐seq study of the murine limbus, Altshuler et al.^36^ identified limbal epithelial cells with a different signature, where the ‘inner’ LSC were actively proliferating and helped renew the cornea epithelium and the ‘outer’ LSC were mainly quiescent. In this study, we used scRNA‐seq to characterize cellular heterogeneity within the LSC population. We found diverse cellular states in vitro among LSC that were assumed to be identical and a clear correlation between proliferation and differentiation or senescence. We also identified FEZ1 and DKK1 as two pivotal regulators of LSC proliferation. To examine discrepancies between in vivo and in vitro findings, the expression levels of both genes were validated in human tissues and corneal wound healing models. DKK1 and FEZ1 were mainly expressed in the limbal region but not in the differentiated corneal epithelium. This centripetal pattern of decreasing expression has also been observed for other genes essential for the human cornea, including p63 and Lrig1.^37^, ^38^, ^39^

FEZ1 has been reported to be a multifunctional protein. Previous data demonstrated that FEZ1 participated in axon outgrowth, fasciculation, microtubular transport, and innate‐immune pathways^40^, ^41^, ^42^, ^43^ but has yet to be associated with stem cell proliferation. Moreover, we speculate that FEZ1 may also promote the proliferation of other types of cells, especially epithelial stem cells. The discovery of effective regulatory factors that can help maintain the proliferative capacity of stem cells will greatly benefit clinical applications. Patients with diabetes have corneal complications such as markedly delayed wound healing caused by defective epithelial cell proliferation, which can further lead to neovascularization and corneal opacity.^44^ Transplanting cultured LSC is an effective stem cell‐based treatment for corneal blindness caused by alkali burns,^11^ and enhancing LSC proliferation could ensure that sufficient transplantable cells are obtained *in vitro*. The FEZ1–DKK1 axis is therefore a promising target for treating the aforementioned clinical conditions, especially for the secreted protein, DKK1, which can be exogenously administered. It is yet to unveil how FEZ1 interacts with DKK1 and influences its expression. Given the fact that FEZ1 exhibits a mild expression in the nucleus of LSC, it is likely that FEZ1 enhanced the DKK1 transcription initiation indirectly by binding and impacting other transcription regulators.

Senescence is a complex physiological process with phenotypes that can be characterized by several traits including cell cycle arrest, morphological alterations, and the secretion of SASPs.^45^ Increasing cell size leads to cytoplasm dilution (proteins and mRNA) and alteration of the DNA:cytoplasm ratio, which impairs maintenance of optimal cell function and contributes to cellular senescence.^46^, ^47^ Our study indicated that *shFEZ1*‐ and *shDKK1* could result in cell volume increase, suggesting that this morphological alteration potentially contributes to cellular senescence in LSC. Of note, senescence in vitro is different from that in vivo in some aspects. After serial passages, in vitro‐cultured cells exhibit a senescence phenotype triggered by enzymatic‐dispersion stress and cumulative damage, without inducing organismal aging. In addition, owing to architectural restrictions, in vitro senescence‐associated morphological alterations (such as an increased cell size) are rarely detectable in vivo.^48^ Thus, although knocking down FEZ1 and DKK1 induced an obvious senescence phenotype in cultured LSC, the corresponding phenotype was not unveiled in vivo, which made the senescence research less comprehensive in this study. To clarify LSC senescence in vivo, both natural and transgenic aging animal models, live tracking probes, and ex vivo‐assessment techniques should be included in future studies.

## AUTHOR CONTRIBUTIONS

Liqiong Zhu and Li Wang performed the experiments and worked together to generate the figures and write the manuscript. Dongmei Liu and Chaoqun Chen participated in most of experiments. Kunlun Mo carried out the animal experiments. Xihong Lan assisted with the CFSE assay. Jiafeng Liu, Ying Huang, and Dianlei Guo provided animals. Mingsen Li, Huaxing Huang, Huizhen Guo, Jieying Tan, and Kang Zhang assisted with the experiments. Hong Ouyang, Jin Yuan, and Jianping Ji proposed the conception, designed the study, and wrote the manuscript.

## CONFLICT OF INTEREST STATEMENT

The authors declare no conflict of interest.

## Supporting information


**FIGURE S1.** Subgroups correlation and pseudotime trajectory analysis of Single‐cell RNA sequencing in cultured LSC. (A) Balloon plot showing the cell count in each cell cluster. (B) Whole‐transcriptome expression and grouped correlation heatmap. (C) GO analysis result of marker genes in Cluster C3 and C5. (D) Monocle pseudotemporal trajectory and clusters facet plots. (E) Monocle trajectory mapping of proliferative and epithelial differentiation genes including MKI67, TOP2A, CCNA2, CCNB2, KRT3 and KRT12. (F) Ridge plot showing the distribution of cell count in each cluster along the monocle pseudotemporal trajectory. (G) PHATE pseudotemporal trajectory and clusters facet plots. (H) PHATE trajectory mapping of proliferative and epithelial differentiation genes.
**FIGURE S2.** Monocle trajectory branch analysis of Single‐Cell RNA sequencing in cultured LSC. (A) Dynamics expression of ordering genes along Monocle pseudotime trajectory by heatmap, and GO analysis of cluster1 and cluster2 genes. (B) Dynamics expression of branching genes uncovered by BEAM and shown by heatmap, and GO analysis of branching genes cluster1 and cluster4. (C) Overlapping of marker genes of single‐cell cluster C3 and branching genes cluster3. (D) GO analysis result of genes overlapped in (C).
**FIGURE S3.** Characterisation of human LSC.(A) Immunostaining images and quantification of LSC specific markers PAX6 and p63 in passage 1 and passage 5 LSC. Phase‐contrast images of LSC (left panel). Scale bars, 200 μm (phase‐contrast images); 50 μm (immunostaining images).
**FIGURE S4.** Cell–cell communication in clusters of cultured LSC scRNA‐seq. (A) Circle plot showing the interaction number and strength in cell clusters. (B) Scatterplot showing the outgoing and incoming interaction strength in cell clusters. (C) Sankey diagram showing the communication patterns and patterns related genes. (D) Heatmap depicting patterns related genes' expression. (E) Heatmap depicting the role of each cell cluster as WNT or FGF signalling sender, receiver, mediator or influencer. (F, I) Circle plots showing the WNT or FGF signalling interactions and strengths among cell clusters. (G, J) Hierarchical plot showing the inferred WNT or FGF signalling communication network. (H, K) Chord diagram showing influenced WNT or FGF signalling L–R pairs among cell clusters.
**FIGURE S5.** Co‐descending genes analysis in both *FEZ1* and *DKK1* depletion LSC. (A) Venn diagram showing the overlapping downregulated genes in *shFEZ1‐* and *shDKK1‐* transfected LSC (left). GO BP analysis result of the co‐descending genes (right). (B) Quantification of AURKB, FGFBP1, FOSL1, FOXM1, HMGA2, HMGB2, and MELK expression level in *scramble*, *shFEZ1‐* and *shDKK1‐* transfected LSC.
**FIGURE S6.** Corneal wound healing model.(A) Representative images of the corneal epithelium treated with FEZ1 antibody. White light micrograph (upper panel), fluorescein staining images (lower panel). (B) H&E staining images of the corneas treated with IgG (as control), FEZ1 antibody, FEZ1 antibody combined with rmDKK1 at 7 days after injury. Scale bars, 100 μm. (C) Quantification of the thickness of corneal epithelium in each group (*n* = 4).

**TABLE S1.** List of reagents
**TABLE S2.** List of shRNA sequences
**TABLE S3.** List of primers
**TABLE S4.** List of antibodies

## Data Availability

The datasets in this study are available from the corresponding author upon reasonable request.
