# Supplementary material for: Single‐cell transcriptomics implicates the FEZ1–DKK1 axis in the regulation of corneal epithelial cell proliferation and senescence
Source: Cell Prolif. 2023 Feb 27;56(9):e13433. doi: 10.1111/cpr.13433 (PMC10472519; doi:10.1111/cpr.13433)
Supplement: Supplementary file 1 — FIGURE S1. Subgroups correlation and pseudotime trajectory analysis of Single‐cell RNA sequencing in cultured LSC. (A) Balloon plot showing the cell count in each cell cluster. (B) Whole‐transcriptome expression and grouped correlation heatmap. (C) GO analysis result of marker genes in Cluster C3 and C5. (D) Monocle pseudotemporal trajectory and clusters facet plots. (E) Monocle trajectory mapping of proliferative and epithelial differentiation genes including MKI67, TOP2A, CCNA2, CCNB2, KRT3 and KRT12. (F) Ridge plot showing the distribution of cell count in each cluster along the monocle pseudotemporal trajectory. (G) PHATE pseudotemporal trajectory and clusters facet plots. (H) PHATE trajectory mapping of proliferative and epithelial differentiation genes. FIGURE S2. Monocle trajectory branch analysis of Single‐Cell RNA sequencing in cultured LSC. (A) Dynamics expression of ordering genes along Monocle pseudotime trajectory by heatmap, and GO analysis of cluster1 and cluster2 genes. (B) Dynamics expression of branching genes uncovered by BEAM and shown by heatmap, and GO analysis of branching genes cluster1 and cluster4. (C) Overlapping of marker genes of single‐cell cluster C3 and branching genes cluster3. (D) GO analysis result of genes overlapped in (C). FIGURE S3. Characterisation of human LSC.(A) Immunostaining images and quantification of LSC specific markers PAX6 and p63 in passage 1 and passage 5 LSC. Phase‐contrast images of LSC (left panel). Scale bars, 200 μm (phase‐contrast images); 50 μm (immunostaining images). FIGURE S4. Cell–cell communication in clusters of cultured LSC scRNA‐seq. (A) Circle plot showing the interaction number and strength in cell clusters. (B) Scatterplot showing the outgoing and incoming interaction strength in cell clusters. (C) Sankey diagram showing the communication patterns and patterns related genes. (D) Heatmap depicting patterns related genes' expression. (E) Heatmap depicting the role of each cell cluster as WNT or FGF [file CPR-56-e13433-s001.docx]

**SUPPLEMENTARY FIGURES**


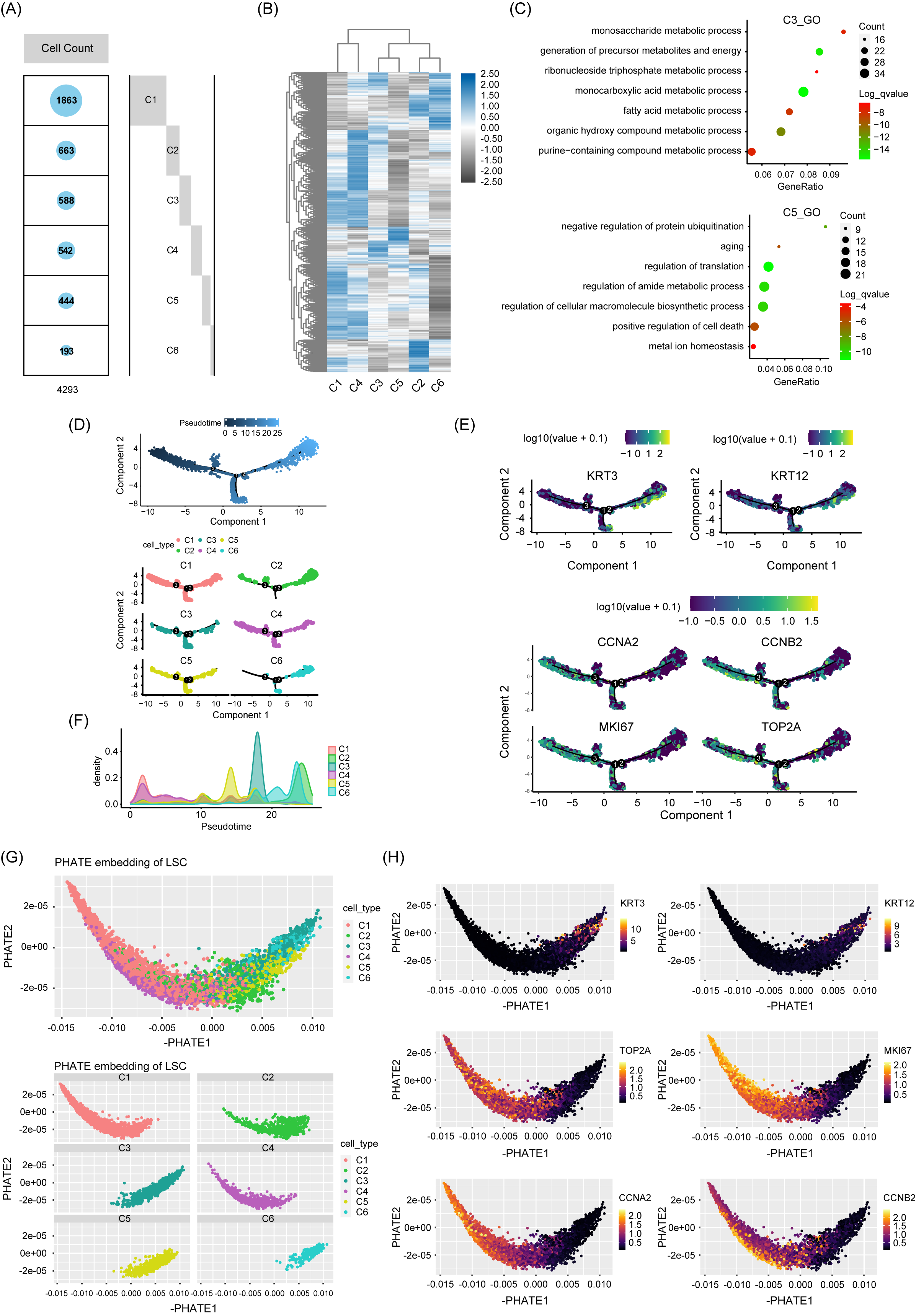


**FIGURE S1.** Subgroups correlation and pseudo-time trajectory analysis of Single-cell RNA sequencing in cultured LSC.

(A) Balloon plot showing the cell count in each cell cluster. (B) Whole transcriptome expression and grouped correlation heatmap. (C) GO analysis result of marker genes in Cluster C3 and C5. (D) Monocle pseudo-temporal trajectory and clusters facet plots. (E) Monocle Trajectory mapping of proliferative and epithelial differentiation genes including MKI67, TOP2A, CCNA2, CCNB2, KRT3 and KRT12. (F) Ridge plot showing the distribution of cell count in each cluster along the monocle pseudo-temporal trajectory. (G) PHATE pseudo-temporal trajectory and clusters facet plots. (H) PHATE trajectory mapping of proliferative and epithelial differentiation genes.


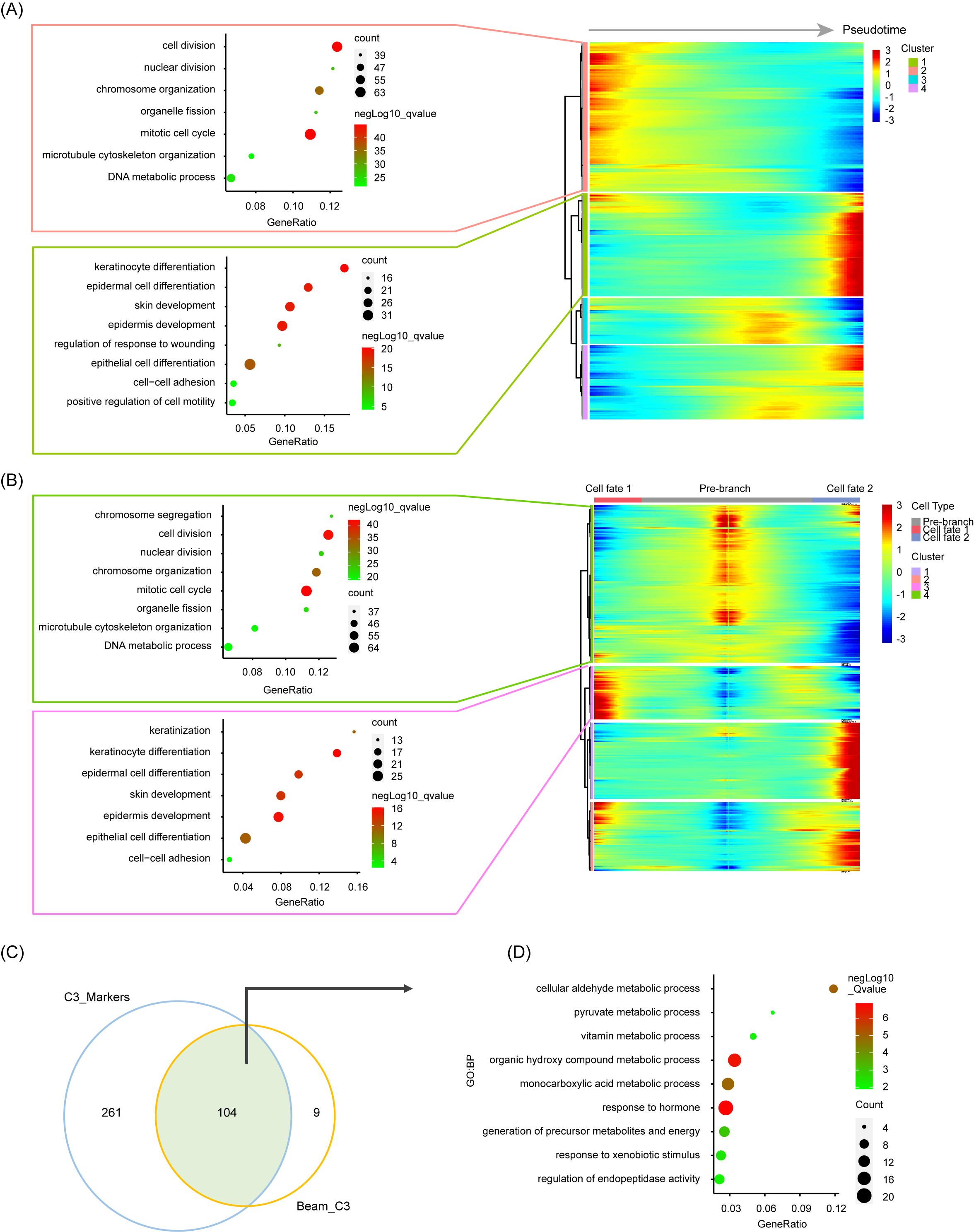


**FIGURE S2.** Monocle trajectory branch analysis of Single-Cell RNA sequencing in cultured LSC.

(A) Dynamics expression of ordering genes along Monocle pseudotime trajectory by heatmap, and GO analysis of cluster1 and cluster2 genes. (B) Dynamics expression of branching genes uncovered by BEAM and shown by heatmap, and GO analysis of branching genes cluster1 and cluster4. (C) Overlapping of marker genes of single cell cluster C3 and branching genes cluster3. (D) GO analysis result of genes overlapped in (C).


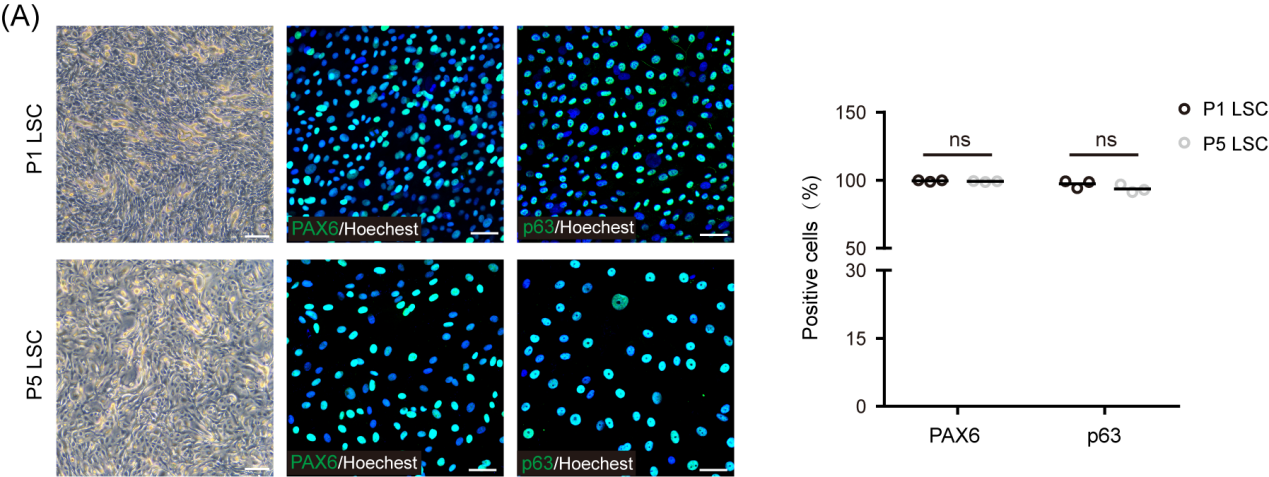


**FIGURE S3.** Characterization of human LSC.

(A) Immunostaining images and quantification of LSC specific markers PAX6 and p63 in passage 1 and passage 5 LSC. Phase-contrast images of LSC (left panel). Scale bars, 200 μm (phase-contrast images); 50 μm (immunostaining images).


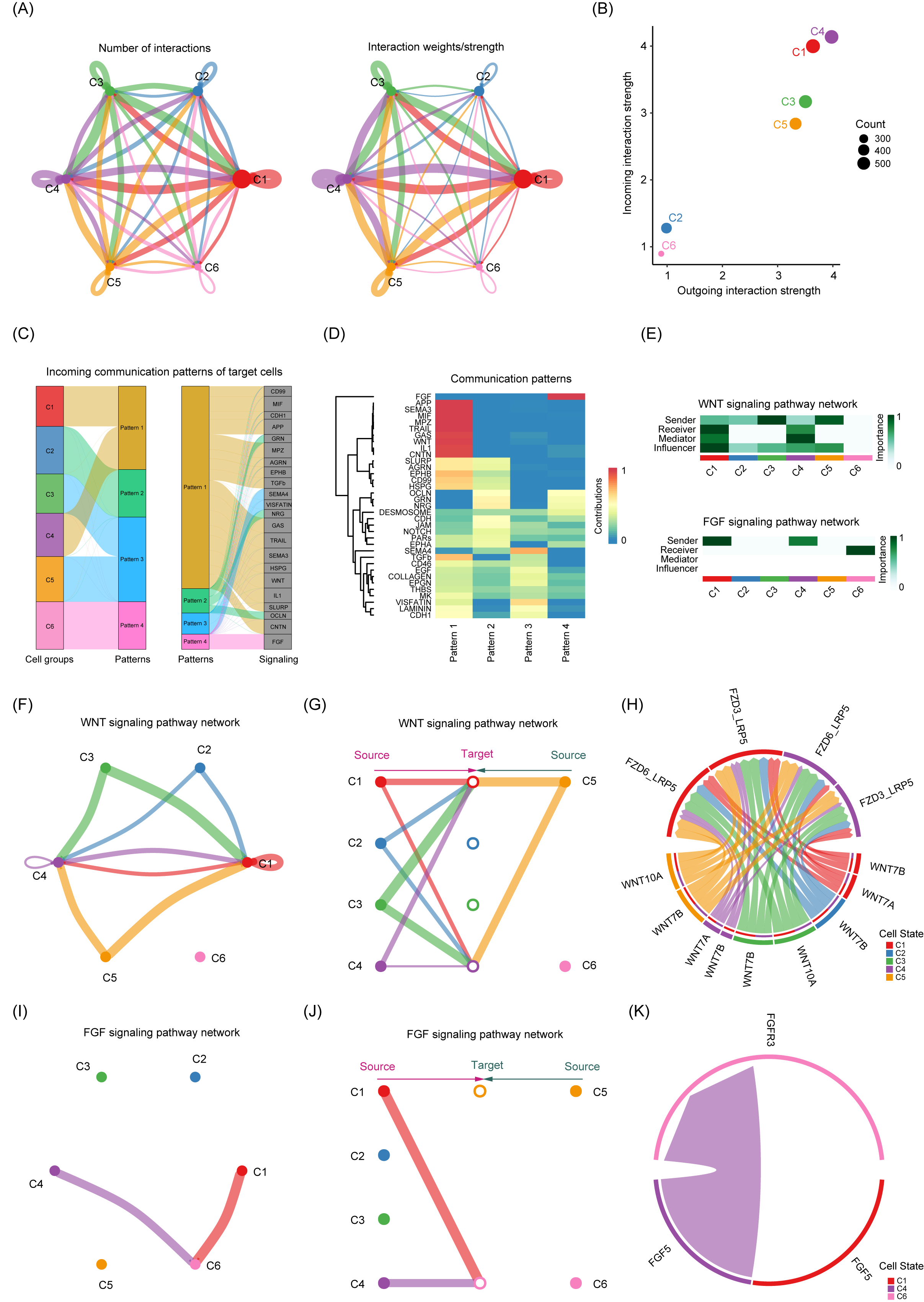


**FIGURE S4.** Cell-cell communication in clusters of cultured LSC scRNA-seq.

(A) Circle plot showing the interaction number and strength in cell clusters. (B) Scatterplot showing the outgoing and incoming interaction strength in cell clusters. (C) Sankey diagram showing the communication patterns and patterns related genes. (D) Heatmap depicting patterns related genes’ expression. (E) Heatmap depicting the role of each cell cluster as WNT or FGF signaling sender, receiver, mediator or influencer. (F, I) Circle plots showing the WNT or FGF signaling interactions and strengths among cell clusters. (G, J) Hierarchical plot showing the inferred WNT or FGF signaling communication network. (H, K) Chord diagram showing influenced WNT or FGF signaling L–R pairs among cell clusters.


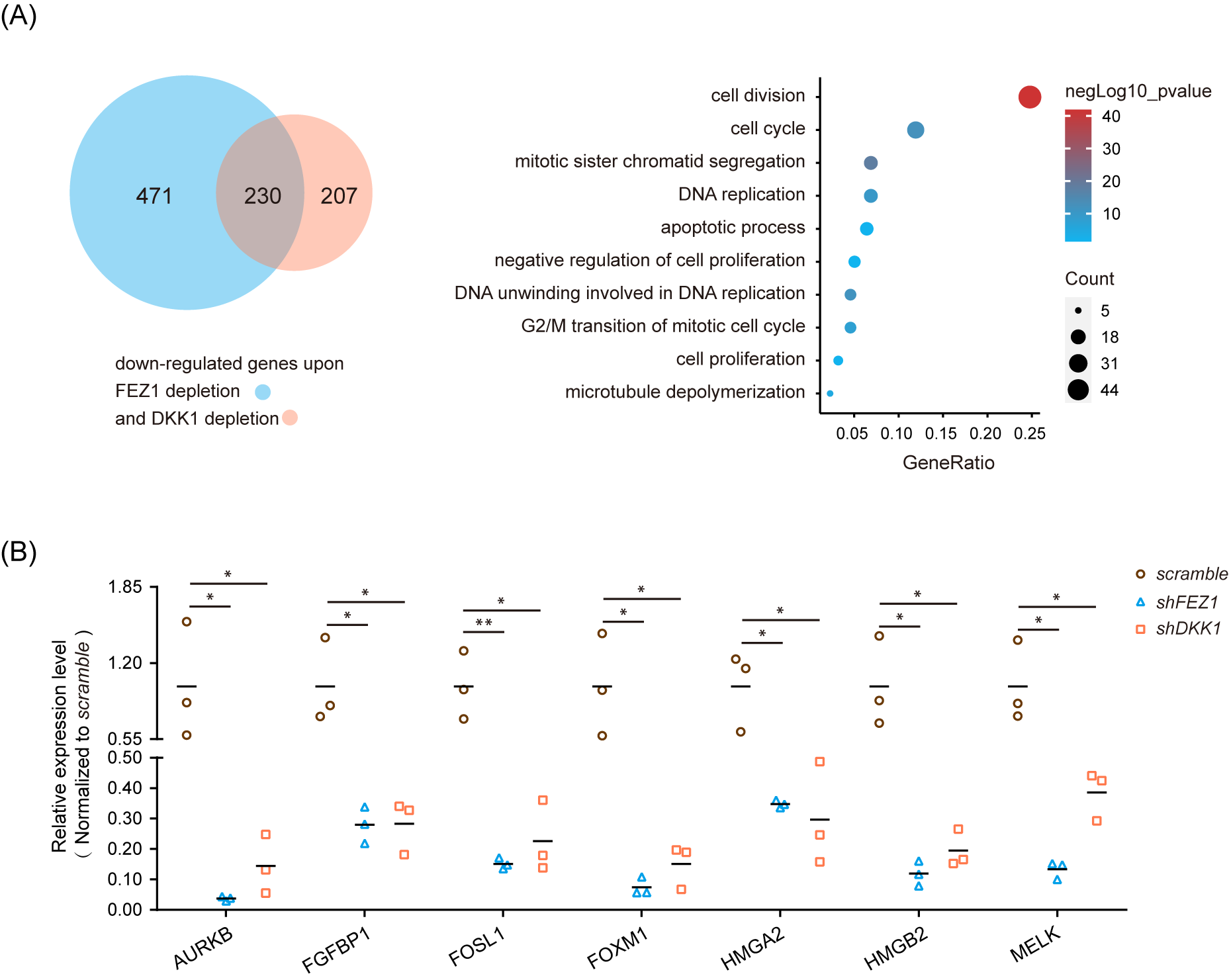


**FIGURE S5.** Co-descending genes analysis in both *FEZ1* and *DKK1* depletion LSC.

1. Venn diagram showing the overlapping down-regulated genes in *shFEZ1-* and *shDKK1-* transfected LSC (left). GO BP analysis result of the co-descending genes (right). (B) Quantification of AURKB, FGFBP1, FOSL1, FOXM1, HMGA2, HMGB2, and MELK expression level in *scramble*, *shFEZ1-* and *shDKK1-* transfected LSC.


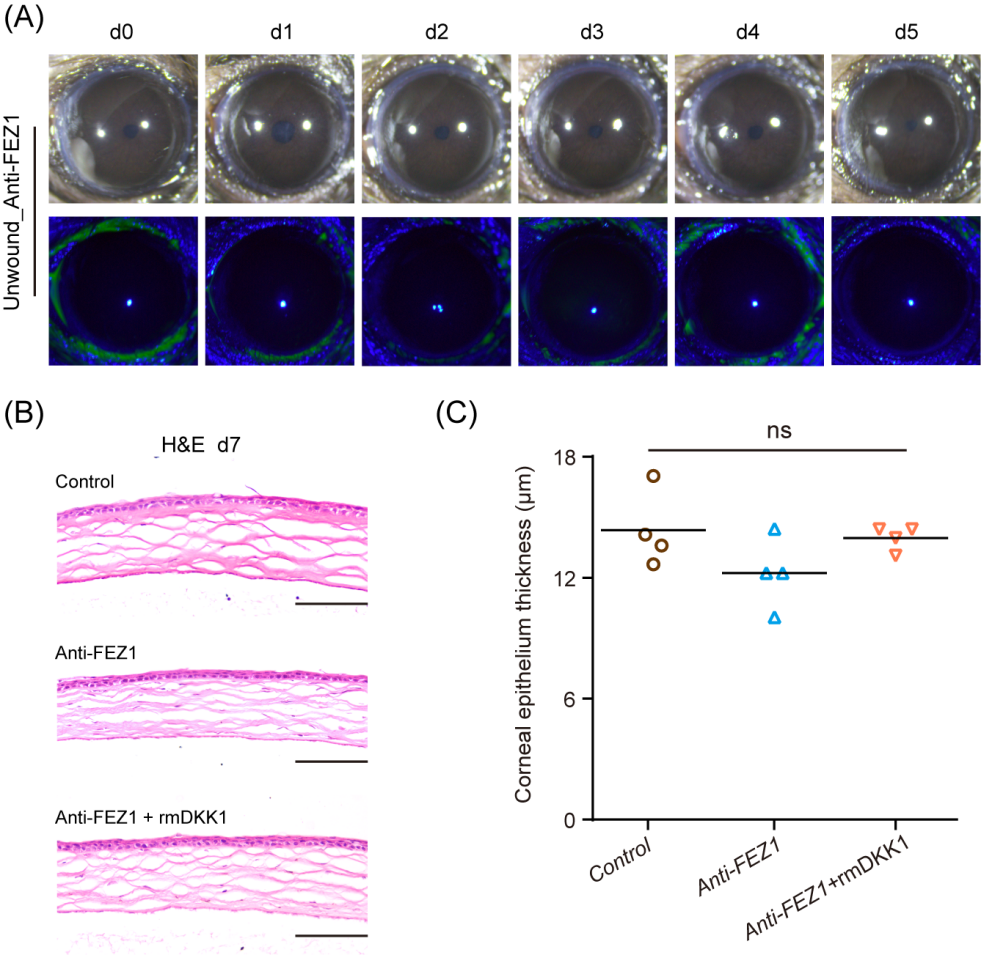


**FIGURE S6.** Corneal wound healing model.

1. Representative images of the corneal epithelium treated with FEZ1 antibody. White light micrograph (upper panel), fluorescein staining images (lower panel). (B) H&E staining images of the corneas treated with IgG (as control), FEZ1 antibody, FEZ1 antibody combined with rmDKK1 at 7 days after injury. Scale bars, 100 μm. (C) Quantification of the thickness of corneal epithelium in each group (*n* = 4).
