# Supplementary material for: Single‐cell transcriptomics implicates the FEZ1–DKK1 axis in the regulation of corneal epithelial cell proliferation and senescence
Source: Cell Prolif. 2023 Feb 27;56(9):e13433. doi: 10.1111/cpr.13433 (PMC10472519; doi:10.1111/cpr.13433)
Supplement: Supplementary file 2 — TABLE S1. List of reagents TABLE S2. List of shRNA sequences TABLE S3. List of primers TABLE S4. List of antibodies [file CPR-56-e13433-s002.docx]

**SUPPLEMENTARY TABLES**

**Supplementary Table S1. List of reagents**

| **Reagents for cell culture** | **Company** | **Cat. No.** |  |
| --- | --- | --- | --- |
| DMEM | Corning | 10-017-CV |  |
| Fetal Bovine Serum (FBS) | Gibco | 10270-106 |  |
| Penicillin/streptomycin | Gibco | 15140-122 |  |
| Type IV collagenase | Gibco | 17104019 |  |
| 0.25% Trypsin-EDTA | ThermoFisher | 25200072 |  |
| collagen I | Gibco | C3867 |  |
| Matrigel | BD biosciences | 354230 |  |
| Recombinant human DKK1(rhDKK1) | MedChemExpress | HY-P7155A |  |
| Lipofectamine 3000 Transfection Kit | Invitrogen | L3000-015 |  |
| Polybrene | Sigma | H9268-5G |  |
| Puromycin | Gibco | A11138-03 |  |
| **Reagents for animal experiment** | **Company** | **Cat. No.** | **Concentration** |
| FEZ1 antibody | Proteintech | 12100-1-AP | 0.2 ug/uL |
| Normal Mouse IgG | EMD Millipore | 12-371 | 0.2 ug/uL |
| Recombinant mouse DKK1(rmDKK1) | MedChemExpress | HY-P7154 | 10 ng/uL |
| **Reagents** | **Company** | **Cat. No.** | **Concentration** |
| Crystal Violet Stain Solution (1%) | Solarbio | G1062 | 0.5% |
| CFSE Cell Proliferation Kit | ABP Biosciences | A001-A | 3uM |
| MitoTracker® Red CMXRos | Cell Signaling Technology | 9082S | 50nM |
| Endo-free Plasmid Mini Kit II | Omega | D6950-01B |  |
| RNA Easy Fast Tissue/Cell Kit | Tiangen | DP451 |  |
| PrimeScript™ RT Master Mix (Perfect Real Time) | Takara | RR036B |  |
| iTaq^TM^ Universal SYBR Green Supermix Kit | Bio-rad | 1725124 |  |
| Senescence β-Galactosidase Staining Kit | Beyotime | C0602 |  |
| Cell Cycle and Apoptosis Analysis Kit | Beyotime | C1052 |  |

**Supplementary Table S2. List of shRNA sequences**

| **shRNA** | **Sequences (5' To 3')** |
| --- | --- |
| scramble | 5′-CCTAAGGTTAAGTCGCCCTCG-3′ |
| shFEZ1-1 | 5′-GCTCAATGTCTGCTTTCGGAA-3′ |
| shFEZ1-2 | 5′-TCCATGGAGGACCTCGTAAAT-3′ |
| shDKK1-1 | 5′-CCAGAAGAACCACCTTGTCTT-3′ |
| shDKK1-2 | 5′-TGTTATCTTGACTGACAAATA-3′ |

**Supplementary Table S3. List of primers**

| **Human primers** |  | **Sequences (5' To 3')** |
| --- | --- | --- |
| KRT12 | Sense | TTCCATGTTTGGTTCTAGTTCCG |
| KRT12 | Antisense | TCATTGCCCGAGAGAATACCTA |
| MKI67 | Sense | GCCTGCTCGACCCTACAGA |
| MKI67 | Antisense | GCTTGTCAACTGCGGTTGC |
| DKK1 | Sense | CCTTGAACTCGGTTCTCAATTCC |
| DKK1 | Antisense | CAATGGTCTGGTACTTATTCCCG |
| FEZ1 | Sense | CCACTGGTGAGTCTGGATGAA |
| FEZ1 | Antisense | CGGAAGAAAAATTCTCAAGCTCG |
| LMNB1 | Sense | AAGCATGAAACGCGCTTGG |
| LMNB1 | Antisense | AGTTTGGCATGGTAAGTCTGC |
| p21 | Sense | TGTCCGTCAGAACCCATGC |
| p21 | Antisense | AAAGTCGAAGTTCCATCGCTC |
| PAX6 | Sense | TGGGCAGGTATTACGAGACTG |
| PAX6 | Antisense | ACTCCCGCTTATACTGGGCTA |
| p63 | Sense | GGACCAGCAGATTCAGAACGG |
| p63 | Antisense | AGGACACGTCGAAACTGTGC |
| GAPDH | Sense | CTGGGCTACACTGAGCACC |
| GAPDH | Antisense | AAGTGGTCGTTGAGGGCAATG |

**Supplementary Table S4. List of antibodies**

| **Antibody** | **Host species** | **Dilution** | **Company** | **Cat. No.** |
| --- | --- | --- | --- | --- |
| FEZ1 | Rabbit | 1∶100 | Proteintech | 12100-1-AP |
| DKK1 | Rabbit | 1∶100 | Proteintech | 21112-1-AP |
| MKI67 | Mouse | 1∶200 | Cell Signaling Technology | 9449S |
| Keratin 12 | Rabbit | 1∶100 | Abcam | ab185627 |
| p63 (ΔN) | Rabbit | 1∶200 | Biolegend | 619002 |
| PAX6 | Rabbit | 1∶200 | Biolegend | PRB-278P |
| Lamin B1 (LMNB1) | Rabbit | 1∶10000 | Santa Cruz Biotechnology | sc-374015 |
| Caveolin-1 | Rabbit | 1∶150 | Proteintech | 16447-1-AP |
| p21 (CDKN1A) | Rabbit | 1∶5000 | Abcam | ab109520 |
